# The effect of radiotherapy in liver-confined but non-resectable Barcelona Clinic Liver Cancer stage C large hepatocellular carcinoma

**DOI:** 10.18632/oncotarget.10908

**Published:** 2016-07-28

**Authors:** Hong In Yoon, Inkyung Jung, Kwang-Hyub Han, Jinsil Seong

**Affiliations:** ^1^ Department of Radiation Oncology, Yonsei Cancer Center, Yonsei University College of Medicine, Yonsei University Health System, Seoul, Korea; ^2^ Department of Biostatistics & Medical Informatics, Yonsei University College of Medicine, Seoul, Korea; ^3^ Department of Internal Medicine, Yonsei University College of Medicine, Yonsei University Health System, Seoul, Korea; ^4^ Korean Liver Cancer Study Group, Seoul, Korea

**Keywords:** large hepatocellular carcinoma, radiotherapy, BCLC stage C, median survival time, propensity score matching

## Abstract

**Background and aims:**

Clinical trials to determine the efficacy of radiotherapy (RT) in liver-confined but non-resectable Barcelona Clinic Liver Cancer (BCLC) stage C hepatocellular carcinoma (HCC) are scarce. We aimed to determine the benefit of RT in such tumors and investigated large HCC tumors.

**Methods:**

HCC data from the Korea Central Cancer Registry recorded from 2008 to 2010 were used. A total of 593 patients met our inclusion criteria; 67 were treated with RT while the remainder made up the non-RT group. Fifty-two RT recipients underwent combination treatments within 4 weeks after the first RT treatment, and were defined as the combination RT group. We performed propensity score matching (PSM) to compare the RT or combination RT groups with the non-RT group. The endpoint was overall survival (OS).

**Results:**

Median follow-up time for surviving patients was 48 months. After PSM, there was no difference in OS between the RT and non-RT groups or between the combination RT and non-RT groups. However, the combination RT group had a longer median survival time (MST) (10.7 vs. 6.9 months, respectively). Next, we conducted PSM between the combination RT and non-RT groups in patients with tumor sizes ≥10 cm; MST was significantly longer in the former group (10.1 vs. 5.4 months, respectively; bootstrap 95% confidence interval of the difference in MST: 0.2-11.8).

**Conclusions:**

As a combined modality, RT is a plausible therapeutic option for liver-confined but non-resectable BCLC stage C large HCC patients.

## INTRODUCTION

Currently, the Barcelona Clinic Liver Cancer (BCLC) staging system serves as a major treatment guideline [1]. BCLC stage C (advanced stage) hepatocellular carcinoma (HCC) is highly aggressive, and has a dismal survival rate despite the clinical introduction of sorafenib, a multi-tyrosine kinase inhibitor [2, 3]. BCLC stage C HCC is very heterogeneous, and shows diverse patterns ranging from liver-confined HCC with portal vein tumor thrombosis to extrahepatic disseminated HCC. Due to the aggressiveness and heterogeneity of BCLC stage C HCC, sub-classification and optimal therapeutic approaches require further investigation.

The multimodal approach is a basic oncologic principle that has been successful in most types of locally advanced cancers. It often involves performing chemotherapy and radiotherapy, followed by surgery if possible [4–6]. Some researchers have examined whether the inclusion of RT in the multimodal approach enhances the survival outcome for BCLC stage C HCC [7–9], and the optimal criteria for local RT have also been evaluated [10, 11]. However, clinical trials for high-level evidence to establish the benefit of additional local RT for BCLC stage C HCC are still lacking.

We recently reported that hepatic arterial infusion concurrent chemoradiotherapy could improve survival for patients with locally advanced HCC with good performance and normal liver function when comparing our institution's database to that of the Korean Liver Cancer Study Group (KLCSG) nationwide multicenter HCC cohort treated between 2003 and 2005 [9]. However, this retrospective study compared two independent databases with different treatment periods. Furthermore, patients who underwent sorafenibwere not included because the KLCSG database was constructed before the sorafenib era. Therefore, additional studies are required to confirm our previous finding.

In this study, we investigated the efficacy of RT, as well as combination therapy involving RT, in comparison to other treatments for liver-confined but non-resectable BCLC stage C HCC using a nationwide database constructed from a multi-center HCC cohort that was based on the Korea Central Cancer Registry. We also ascertained the survival benefit of RT in bulky liver-confined but non-resectable BCLC stage C HCC.

## RESULTS

### Patient characteristics and survival analysis before PSM

The characteristics of all the enrolled patients before propensity score matching (PSM) are listed in Table 1. There was a significant difference in performance status (PS), portal vein (PV) invasion, age, tumor size, and α-fetoprotein (AFP) levels between the 2 groups. The median ages of patients in the RT and non-RT groups were 51 (26−83) and 57 (8-98) years, respectively (*P* = 0.001). The RT group had significantly better PS than the non-RT group; however, the RT group exhibited significantly worse prognostic factors than the non-RT group, including larger tumor size, higher AFP levels, and greater PV invasion. The Child Pugh score was not statistically different between the RT and non-RT groups, although there was marginal significance (*P* = 0.077).

**Table 1:** Baseline characteristics for whole cohort

Variables		Radiotherapy (n = 67)		Non-radiotherapy					
				Before PSM (n = 526)			After PSM (n = 67)		
		n	(%)	n	(%)	p	n	(%)	*p*
Age	Median	51		57		0.001	54		0.86
	Range	(26-83)		(8-98)			(8-80)		
Gender	Male	53	(79.1)	440	(83.7)	0.349	53	(79.1)	>0.99
	Female	14	(20.9)	86	(16.3)		14	(20.9)	
Performance status	0	36	(53.7)	196	(37.3)	0.023	32	(47.8)	0.56
	1	15	(22.4)	169	(32.1)		9	(13.4)	
	2	0	(0.0)	26	(4.9)		4	(6.0)	
	Unknown	16	(23.9)	135	(25.7)		22	(32.8)	
Smoking history	No	31	(46.3)	251	(47.7)	0.823	32	(47.8)	0.87
	Yes	36	(53.7)	275	(52.3)		35	(52.2)	
Pack years (yrs)	Median	5		0		0.328	0		0.32
	Range	(0-70)		(0-160)			(0-120)		
Tumor size (cm)	Median	10		6.6		<0.001	10		0.41
	Range	(1.3-16)		(0.8-19.2)			(1.9-19.2)		
AFP	Median	1040.4		228.8		0.043	917		0.6
	Range	(0.8-298937)		(0.8-3004000)			(3.21-134000)		
Child Pugh score	Median	5		6		0.077	5		0.69
	Range	(5-9)		(5-9)			(5-9)		
Child Pugh	A	48	(71.6)	336	(63.9)	0.21	48	(71.6)	>0.99
classification	B	19	(28.4)	190	(36.1)		19	(28.4)	
Growth pattern	Solitary	37	(55.2)	270	(51.3)	0.375	28	(41.8)	0.27
	2	4	(6.0)	69	(13.1)		10	(14.9)	
	3	4	(6.0)	22	(4.2)		1	(1.5)	
	4	0	0.0	9	(1.7)		2	(3.0)	
	≥ 5	22	(32.8)	156	(29.7)		26	(38.8)	
Nodal metastasis	III	59	(88.1)	463	(88.0)	0.993	63	(94.0)	0.22
	IV-A	8	(11.9)	63	(12.0)		4	(6.0)	
Portal vein invasion	Yes	56	(83.6)	319	(60.6)	<0.001	59	(88.1)	0.37
	No	11	(16.4)	207	(39.4)		8	(11.9)	
Hepatic vein invasion	Yes	7	(10.4)	66	(12.5)	0.622	8	(11.9)	0.78
	No	60	(89.6)	460	(87.5)		59	(88.1)	
Bile duct invasion	Yes	5	(7.5)	30	(5.7)	0.579	0	0.0	0.99
	No	62	(92.5)	496	(94.3)		67	(100.0)	
Hepatic artery invasion	Yes	1	(1.5)	7	(1.3)	1	1	(1.5)	>0.99
	No	66	(98.5)	519	(98.7)		66	(98.5)	
The etiology of HCC	HBV & HCV	1	(1.5)	8	(1.5)	0.153	0	0.0	0.94
	HBV	50	(74.6)	348	(66.2)		52	(77.5)	
	HCV	4	(6.0)	37	(7.0)		2	(3.0)	
	Alcohol	3	(4.5)	63	(12.0)		3	(4.5)	
	NBNC	5	(7.4)	17	(3.2)		4	(6.0)	
	Unknown	4	(6.0)	53	(10.1)		6	(9.0)	

Abbreviation: PSM, propensity score matching; AFP, alpha fetoprotein; HCC, hepatocellular carcinoma; HBV, Hepatitis B virus; HCV, Hepatitis C virus; NBNC, non-HBV and non-HCV

**Figure 1:**
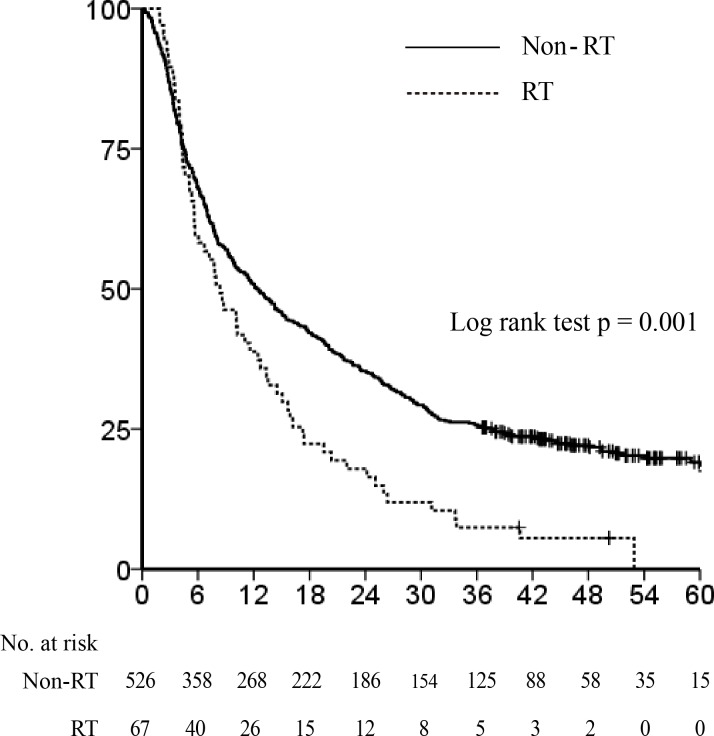
Overall survival before propensity score matching The Kaplan-Meier survival curve shows that the non-radiotherapy (RT) group showed significantly longer overall survival than the RT group (median survival time 12.3 *vs*. 8.4 months, *P* = 0.001).

Median follow-up time for surviving patients was 48 months (range, 36.6-72.5 months). Before PSM, Kaplan-Meier analyses demonstrated that the non-RT group exhibited significantly longer overall survival (OS) than the RT group (median survival time [MST] 12.3 *vs*. 8.4 months respectively, *P* = 0.001, Figure 1). Because the RT group showed more adverse prognostic factors than the non-RT group, we performed PSM analysis to adjust for the significant differences in baseline characteristics between the 2 groups.

### RT *versus* non-RT group outcomes after PSM

**Figure 2:**
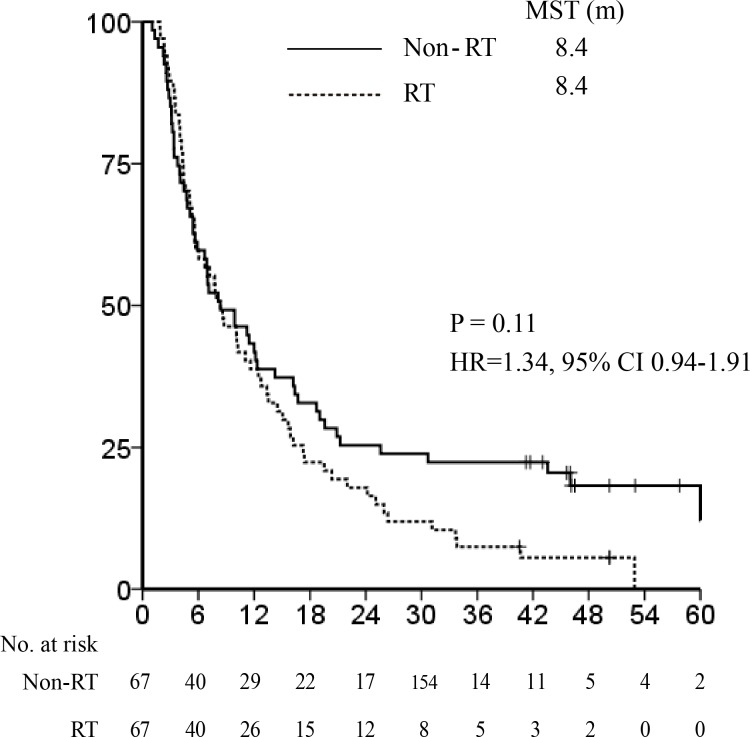
Median survival time after propensity score matching: radiotherapy *vs* non-radiotherapy groups. The Kaplan-Meier survival curve in the entire cohort shows that the median survival time (MST) of the radiotherapy (RT) group (8.4 months) was not significantly different from that of non-RT group (8.4 months; hazard ratio 1.34, 95% confidence interval 0.94-1.91, *P* = 0.11).

After PSM between the RT (*n* = 67) and non-RT (*n* = 526) groups, all clinical factors were evenly distributed between each group (Table 1). Survival curves indicated that the MST of the RT group was not significantly different from that of the non-RT group (8.4 *vs*. 8.4 months, hazard ratio [HR] 1.34, 95% confidence interval (CI) 0.94-1.91, *P* = 0.11, Figure 2).

### Combination RT *versus* non-RT groups after PSM analysis

**Figure 3:**
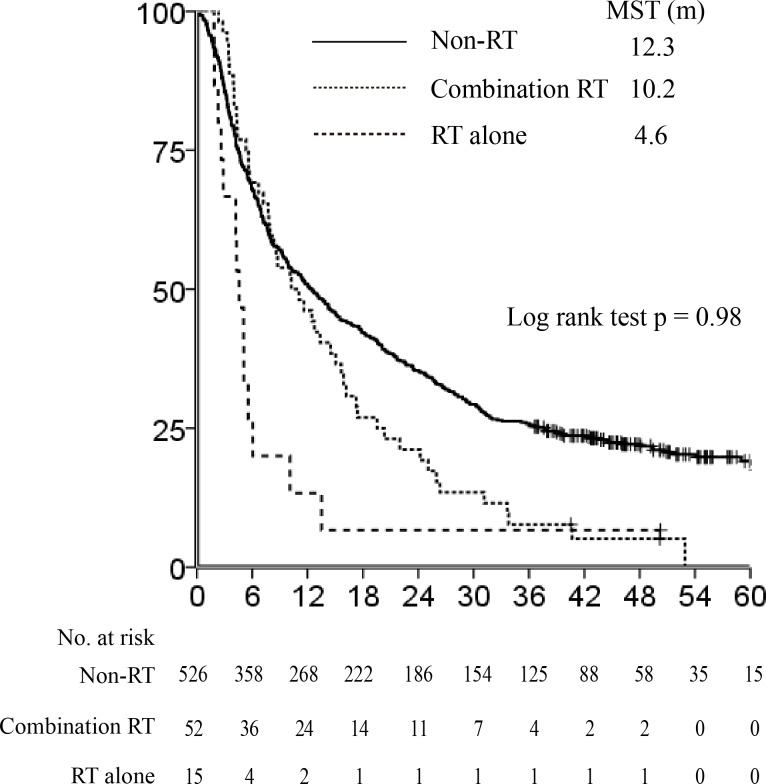
Difference of survival outcome between patients who underwent combination radiotherapy (RT) and RT alone The non-RT and combination RT groups had significantly longer median survival times (MST) than the RT-alone group (12.3 *vs*. 10.2 *vs*. 4.6 months, respectively, *P* = 0.0002).

**Figure 4:**
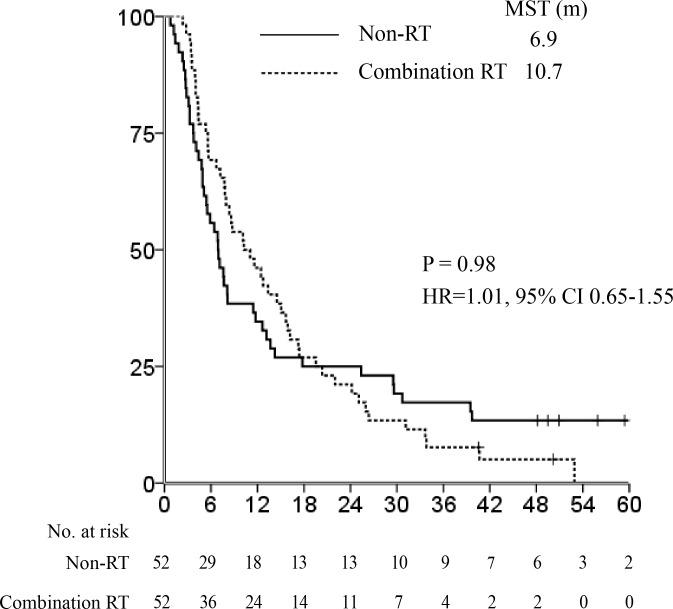
Median survival time after propensity score matching: combination radiotherapy *vs* non-radiotherapy groups. The survival outcomes in the combination radiotherapy (RT) and non-RT groups were not statistically different, although the combination RT group had a longer median survival time (MST) (10.7 *vs*. 6.9 months respectively, hazard ratio 1.01, 95% confidence interval 0.65-1.55, *P* = 0.98).

As described above, the RT group included patients who underwent RT alone (*n* = 15) and in combination with other treatments *n* = 52). We compared the OS in the RT alone, combination RT, and non-RT groups before PSM. Figure 3 shows that the non-RT and combination RT groups had significantly longer MSTs than the RT alone group (12.3 *vs*. 10.2 *vs*. 4.6 months, respectively; *P* = 0.0002). Thus, we performed a second PSM analysis between the combination RT and non-RT groups to verify the effect of RT when combined with other treatments (Table 2). Before PSM analysis, there was a significant difference in PS, PV invasion, age, tumor size, Child Pugh score, and Child Pugh classification between the 2 groups. After PSM analysis using covariates that showed substantial differences between the combination RT and non-RT groups, all clinical factors were distributed evenly. In this case, the survival outcomes of the combination RT and non-RT groups were not significantly different, although the former group had a longer MST (10.7 *vs*. 6.9 months, respectively; HR 1.01, 95% CI 0.65-1.55, *P* = 0.98, Figure 4).

**Table 2:** Baseline characteristics between combination RT and non-RT groups for whole cohort

Variables		Radiotherapy (n = 52)		Non-radiotherapy					
				Before PSM (n = 526)			After PSM (n = 52)		
		n	(%)	n	(%)	*p*	n	(%)	*p*
Age	Median	51		57		0.001	52		0.55
	Range	(26-83)		(8-98)			(35-74)		
Gender	Male	43	(82.7)	440	(83.7)	0.859	47	(90.4)	0.290
	Female	9	(17.3)	86	(16.3)		5	(9.6)	
Performance status	0	31	(59.6)	196	(37.3)	0.011	30	(57.7)	>0.99
	1	10	(19.2)	169	(32.1)		10	(19.2)	
	2	0	(0.0)	26	(4.9)		1	(1.9)	
	Unknown	11	(21.2)	135	(25.7)		11	(21.2)	
Smoking history	No	21	(40.4)	251	(47.7)	0.312	22	(42.3)	0.86
	Yes	31	(59.6)	275	(52.3)		30	(57.7)	
Pack years (yrs)	Median	8		0		0.087	10		0.86
	Range	(0-70)		(0-160)			(0-90)		
Tumor size (cm)	Median	10		6.6		<0.001	10		0.59
	Range	(2-16)		(0.8-19.2)			(1.9-19.2)		
AFP	Median	1024.1		228.8		0.153	697		0.35
	Range	(0.8-200000)		(0.8-3004000)			(1.02-134000)		
Child Pugh score	Median	5		6		0.004	5		0.68
	Range	(5-8)		(5-9)			(5-8)		
Child Pugh	A	42	(80.8)	336	(63,9)	0.015	42	(80.8)	>0.99
classification	B	10	(19.2)	190	(36.1)		10	(19.2)	
Growth pattern	Solitary	30	(57.7)	270	(51.3)	0.353	30	(57.6)	0.77
	2	3	(5.8)	69	(13.1)		2	(3.9)	
	3	4	(7.7)	22	(4.2)		2	(3.9)	
	4	0	0.0	9	(1.7)		0	(3.0)	
	≥ 5	15	(28.8)	156	(29.7)		18	(34.6)	
Nodal metastasis	III	45	(86.5)	463	(88.0)	0.754	51	(98.1)	0.07
	IV-A	7	(13.5)	63	(12.0)		1	(1.9)	
Portal vein invasion	Yes	44	(84.6)	319	(60.6)	0.001	45	(86.5)	0.76
	No	8	(15.4)	207	(39.4)		7	(13.5)	
Hepatic vein invasion	Yes	5	(9.6)	66	(12.5)	0.539	8	(15.4)	0.33
	No	47	(90.4)	460	(87.5)		44	(84.6)	
Bile duct invasion	Yes	3	(5.8)	30	(5.7)	>0.99	3	(5.8)	>0.99
	No	49	(94.2)	496	(94.3)		49	(94.2)	
Hepatic artery invasion	Yes	1	(1.9)	7	(1.3)	1	0	0.0	0.99
	No	51	(98.1)	519	(98.7)		52	(100.0)	
The etiology of HCC	HBV & HCV	1	(1.9)	8	(1.5)	0.175	2	(3.9)	0.97
	HBV	36	(69.2)	348	(66.2)		39	(75.0)	
	HCV	4	(7.7)	37	(7.0)		5	(9.6)	
	Alcohol	3	(5.8)	63	(12.0)		2	(3.9)	
	NBNC	5	(9.6)	17	(3.2)		1	(1.9)	
	Unknown	3	(5.8)	53	(10.1)		3	(5.7)	

Abbreviation: PSM, propensity score matching; AFP, alpha fetoprotein; HCC, hepatocellular carcinoma; HBV, Hepatitis B virus; HCV, Hepatitis C virus; NBNC, non-HBV and non-HCV

### OS in combination RT *versus* non-RT groups after PSM analysis in patients with tumor sizes ≥10 cm

**Figure 5:**
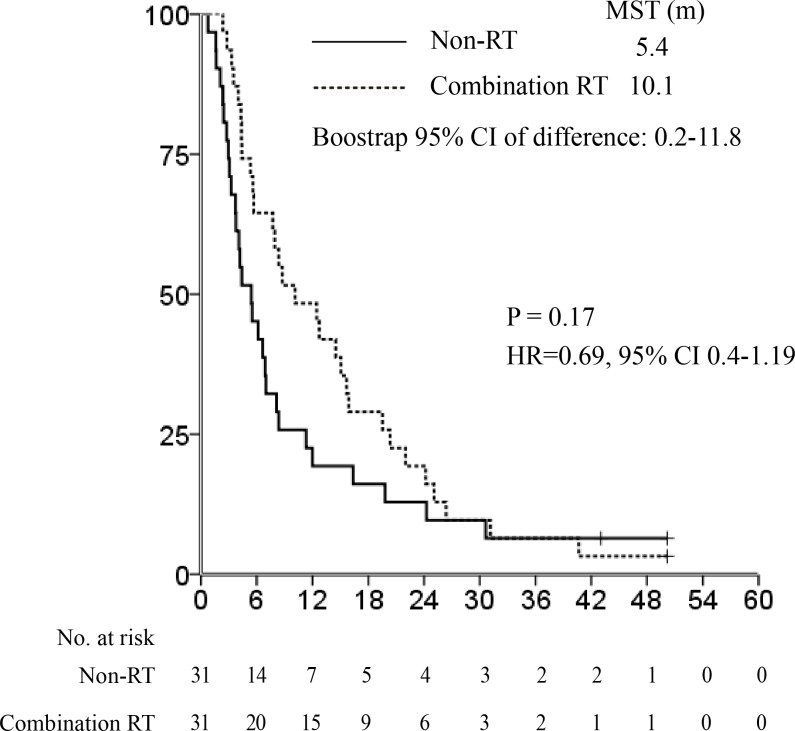
Median survival time after propensity score matching: combination radiotherapy *vs* non-radiotherapy groups in the subset of patients with tumor sizes ≥10 cm. There was a significant difference in median survival time (MST) between the 2 groups (10.1 *vs*. 5.4 months, bootstrap 95% confidence interval [CI] of difference in MST, 0.2-11.8). The combination RT group achieved better overall survival despite no statistical significance (hazard ratio 0.69, 95% CI 0.4-1.19, *P* = 0.17).

Subsequently, we performed PSM between the patients in the combination RT and non-RT groups with tumor sizes ≥10 cm (*n* = 31 and *n* = 169, respectively) to identify the survival benefit of RT in liver-confined bulky non-resectable tumors. There was a significant difference in age and tumor size between the 2 groups before PSM (Table 3). PV invasion and HCC etiology were also significantly different between the groups (*P* = 0.072 and *P* = 0.06, respectively). Thus, we performed PSM *via* logistic regression using age, tumor size, PV invasion, and HCC etiology; all patient characteristics were balanced after PSM analysis. On survival analysis after PSM, we observed a significant difference in MST between the 2 groups (10.1 *vs*. 5.4 months respectively, bootstrap 95% CI of difference in MST, 0.2-11.8; Figure 5). The combination RT group achieved better OS, although the difference was not statistically significant (HR 0.69, 95% CI 0.4-1.19, *P* = 0.17; Figure 5).

**Table 3:** Baseline characteristics between combination RT and non-RT groups in subgroup of tumor size ≥ 10 cm

Variables		Radiotherapy (n = 31)		Non-radiotherapy					
				Before PSM (n = 169)			After PSM (n = 31)		
		n	(%)	n	(%)	*p*	n	(%)	*p*
Age	Median	51		53		0.045	52		0.66
	Range	(26-83)		(8-85)			(33-80)		
Gender	Male	25	(80.7)	145	(85.8)	0.424	24	(77.4)	>0.99
	Female	6	(19.3)	24	(14.2)		7	(22.6)	
Performance status	0	16	(51.6)	76	(45.0)	0.575	15	(48.4)	0.56
	1	6	(19.4)	32	(18.9)		5	(16.1)	
	2	0	(0.0)	4	(2.4)		1	(3.2)	
	Unknown	9	(29.0)	57	(33.7)		10	(32.3)	
Smoking history	No	12	(38.7)	67	(39.6)	0.922	15	(48.4)	0.87
	Yes	19	(61.3)	102	(60.4)		16	(51.6)	
Pack years (yrs)	Median	7		7		0.862	0		0.22
	Range	(0-70)		(0-80)			(0-45)		
Tumor size (cm)	Median	10		10		0.013	10		0.64
	Range	(10-16)		(10-19.2)			(10-17)		
AFP	Median	1020.6		1296.5		0.175	208.5		0.43
	Range	(2.1-200000)		(1.75-3004000)			(2.7-121000)		
Child Pugh score	Median	6		6		0.176	6		0.07
	Range	(5-8)		(5-9)			(5-9)		
Child Pugh	A	24	(77.4)	108	(63.9)	0.144	17	(54.8)	>0.99
classification	B	7	(22.6)	61	(36.1)		14	(45.2)	
Growth pattern	Solitary	17	(54.8)	77	(45.6)	0.625	10	(32.3)	0.27
	2	1	(3.2)	13	(7.7)		2	(6.5)	
	3	1	(3.2)	3	(1.8)		0	0.0	
	4	0	0.0	1	(0.6)		1	(3.2)	
	≥ 5	12	(38.8)	75	(44.4)		18	(58.0)	
Nodal metastasis	III	29	(93.5)	144	(85.2)	0.265	28	(90.3)	0.22
	IV-A	2	(6.5)	25	(14.8)		3	(9.7)	
Portal vein invasion	Yes	28	(90.3)	128	(75.7)	0.072	29	(93.5)	0.37
	No	3	(9.7)	41	(24.3)		2	(6.5)	
Hepatic vein invasion	Yes	4	(12.9)	24	(14.2)	>0.99	4	(12.9)	0.78
	No	27	(87.1)	145	(85.8)		27	(87.1)	
Bile duct invasion	Yes	1	(3.2)	8	(4.7)	>0.99	2	(6.5)	0.99
	No	30	(96.8)	161	(95.3)		29	(93.5)	
Hepatic artery invasion	Yes	1	(3.2)	1	(0.6)	0	0	0.0	>0.99
	No	30	(96.8)	168	(99.4)		31	(100.0)	
The etiology of HCC	HBV & HCV	0	0.0	0	0.0	0.06	0	0.0	0.94
	HBV	22	(71.0)	122	(72.2)		22	(71.0)	
	HCV	2	(6.5)	7	(4.1)		4	(12.9)	
	Alcohol	1	(3.2)	20	(11.8)		1	(3.2)	
	NBNC	4	(12.9)	4	(2.4)		3	(9.7)	
	Unknown	2	(6.5)	16	(9.5)		1	(3.2)	

Abbreviation: PSM, propensity score matching; AFP, alpha fetoprotein; HCC, hepatocellular carcinoma; HBV, Hepatitis B virus; HCV, Hepatitis C virus; NBNC, non-HBV and non-HCV

## DISCUSSION

This nationwide retrospective cohort study utilizing PSM analysis demonstrated that there was no significant difference in OS between the RT and non-RT groups for liver-confined but non-resectable BCLC stage C HCC patients. Furthermore, the OS was not significantly different when comparing the combination RT to the non-RT groups after PSM. However, the combination RT group exhibited longer MST, although without statistical significance. Additionally, we performed PSM between combination RT and non-RT subgroups for patients with tumor sizes ≥10 cm. We found that the combination RT group exhibited significantly improved MST compared to the non-RT group in the subset of patients with larger tumors, and that the combination RT group achieved better OS (although the improvement was not statistically significant).

Our findings are subject to certain limitations; for example, this study was retrospective, and the number of patients included in the RT group was small. The patients were heterogeneous and the proper descriptions of RT modality, total doses and fractionations were lacking. Furthermore, there is no data regarding clinical events and toxicity available through our database. Despite these drawbacks, this study is the first to use a single nationwide multi-center cohort to investigate the survival benefit of RT in liver-confined but non-resectable BCLC stage C HCC patients. Additionally, there was a significant difference in MST between the combination RT and non-RT groups in the subset of patients with bulky tumors.

Large-size HCCs are known to have higher AFP levels, higher rates of microscopic and macroscopic vascular invasion, more advanced stages, and rupturing compared to small HCCs [17, 18]. The prognosis of this disease is dismal, with a 5-year disease-free survival rate of 12.7-38.6% and a 5-year OS rate of 16.7-40.2% despite radical resection [17–21]. However, resectable cases do not account for an appreciable portion of large HCC patients [10]. Several types of treatments have been performed for huge HCC, including trans-arterial therapy, chemotherapy, and RT [22–25]. Recent studies have demonstrated that combining RT with other treatments, such as trans-arterial therapy or hepatic arterial infusion chemotherapy (HAIC), is effective for large HCC [25–27]. Kim et al. reported that RT with concurrent HAIC (HAICCRT) followed by HAIC or RT plus TACE resulted in excellent intrahepatic control and improvement of survival (MST: 12.8 months for HAICCRT and 15.3 months for RT plus TACE) for non-resectable very large HCCs [25]. Zhong et al. investigated the safety and efficacy of stereotactic body radiotherapy combined with TACE for large HCC and reported excellent therapeutic outcomes, with an objective response rate of 79.1% and MST of 12.2 months [26]. Similar to the results of previous studies, our findings indicate that RT combined with another treatment, particularly trans-arterial therapy, could enhance survival. These findings indicate the clinical benefit of choosing RT for large non-resectable HCC as the preferred treatment. In principle, a prospective randomized clinical trial is required to verify our findings regarding the survival benefit of additional RT for large non-resectable HCC.

Our findings demonstrated that the combination RT group achieved better OS in patients with tumor sizes ≥10 cm, although the improvement was not significant. Generally, MST may be more appropriate for evaluating treatment outcomes of patients with poor prognoses. Indeed, significantly longer MST was demonstrated in the combination RT group than in the non-RT group in this study.

In conclusion, this nationwide retrospective cohort study demonstrates that the addition of RT to other treatments is associated significantly with improvement of MST for large HCCs by approximately 5 months. RT as a combined modality can be considered a therapeutic option for liver-confined but non-resectable BCLC stage C large HCC patients.

## METHODS AND MATERIALS

### Multi-center hepatocellular carcinoma cohort

HCC data from the Korea Central Cancer Registry were used to perform the systemic randomized sampling. This database was based on information collected from 47 institutions in Korea; patients enrolled in this database underwent treatment for HCC between 2008 and 2010. This database included information on age; sex; PS; smoking history; pack years; tumor size; AFP levels; Child-Pugh class or score; number of tumor masses; nodal metastasis; PV, hepatic vein, bile duct, and hepatic artery invasion; HCC etiology; and survival. Staging was determined according to the BCLC staging system [1]. HCC was diagnosed according to the practice guidelines for diagnosis and treatment of HCC of the KLCSG [12, 13]. HCC was defined as pathologic confirmation of HCC or compatible radiological findings with the elevation of serum AFP level (> 400 IU/mL before 2009 or > 200 IU/mL after 2009) [12, 13]. If AFP levels were lower than the cut-off values (i.e., ≤400 IU/mL before 2009 or ≤200 IU/mL after 2009), HCC was diagnosed by radiological findings using at least two imaging modalities [12, 13].

### Patient selection and groups

There were 4596 patients in total; inclusion criteria were liver-confined, non-resectable BCLC stage C HCC. We excluded patients according to the following criteria: 1) BCLC stages A, B, and D; 2) distant metastasis; 3) patients who underwent surgery as the first treatment or had no treatment; or 4) missing clinical factor information. Ultimately, 593 patients were included in this study. Sixty-seven patients were treated with RT and defined as the RT group. The remaining patients were considered the non-RT group. In the RT group, 52 (77.6%) patients underwent combination treatments involving other modalities within 4 weeks after the initial treatment; these patients were defined as the combination RT group.

### Treatments

RT, sorafenib, systemic chemotherapy, trans-arterial therapy, and local ablation therapy were performed on patients in this study. Trans-arterial therapy included trans-arterial chemoembolization (TACE) with gelform, trans-arterial chemolipiodolization without gelform, trans-arterial therapy without gelform or lipiodol, TACE with beads, and radioembolization. The information regarding regimens for systemic chemotherapy was not available in the database; however, we expect that most patients in this study were treated with systemic chemotherapy regimens according to the KLCSG practice guidelines for diagnosis and treatment of HCC [12, 13]. Local ablation therapy consisted of radiofrequency ablation, ethanol ablation, and other ablation therapies. For the 52 combination RT patients, the other therapies included sorafenib, systemic chemotherapy, and trans-arterial therapy in 13, 2, and 37 patients, respectively. In the non-RT group, local ablation therapy was administered to 34 patients (6.5%), trans-arterial therapy to 444 patients (84.4%), systemic chemotherapy to 32 patients (6.1%), and sorafenib to 16 patients (3.0%).

### Study design

The study was performed and reported in accordance with the recommendations of the Strengthening the Reporting of Observational Studies in Epidemiology statement [14]. This study was approved by the internal review boards at Severance hospital (IRB no. 4-2015-1157). First, we compared the baseline characteristics between the treatment groups to evaluate the differences in confounding covariates. Next, we performed PSM analysis to adjustment for variables that showed statistically significant differences, and then analyzed the survival difference between treatment groups.

### Statistical analysis

The primary endpoint was OS, which was defined as the period between the treatment start date and the date of death or last follow-up. Before PSM analysis, OS for all patients included in this study was calculated using the Kaplan-Meier method and compared between the RT and non-RT groups using the log-rank test. To minimize potential confounding effects of covariates and selection bias, we utilized PSM analysis for data balancing between groups [15]. For assessment of the difference in potential confounders between the RT and non-RT groups, we performed Pearson's χ^2^ test or Fisher's exact test to compare differences in nominal variables in patient characteristics; differences in continuous variables were analyzed using the Mann-Whitney U test.

We performed 1 to 1 ratio PSM analysis thrice to compare 1) the RT group to the non-RT group, 2) the combination RT group to the non-RT group, and 3) the combination RT group to the non-RT group in the subset of patients with tumor sizes ≥10 cm. After performing each PSM analysis, we compared the baseline characteristics between the 2 groups by using conditional logistic regression. For survival analysis after PSM, we performed Cox regression model analyses to compare the risks of outcome in each group using robust standard errors that considered the clustering of matched pairs. We graphed survival curves using Kaplan-Meier estimates, and compared the OS of treatment groups using the methods devised by Klein and Moeschberger [16].

All statistical analyses were 2-sided. P values ≤0.05 were considered statistically significant. All statistical analyses were conducted using the SAS software, version 9.2.

### Ethics statement

This study was approved by the Institutional Review Board at Severance Hospital. All clinical investigations have been conducted according to the principles expressed in the Declaration of Helsinki.
